# A novel immune-related lncRNA pair signature for prognostic prediction and immune response evaluation in gastric cancer: a bioinformatics and biological validation study

**DOI:** 10.1186/s12935-022-02493-2

**Published:** 2022-02-10

**Authors:** Jun Wang, Beidi Wang, Biting Zhou, Jing Chen, Jia Qi, Le Shi, Shaojun Yu, Guofeng Chen, Muxing Kang, Xiaoli Jin, Lie Wang, Jinghong Xu, Linghua Zhu, Jian Chen

**Affiliations:** 1 Department of Gastroenterology Surgery, The Second Affiliated Hospital, Zhejiang University School of Medicine, Hangzhou, 310000 Zhejiang China; 2 Cancer Institute (Key Laboratory of Cancer Prevention and Intervention, China National Ministry of Education, Key Laboratory of Molecular Biology in Medical Sciences, Zhejiang Province, China), The Second Affiliated Hospital, Zhejiang University School of Medicine, Hangzhou, 310000 Zhejiang China; 3 Department of Colorectal Surgery and Oncology, The Second Affiliated Hospital, Zhejiang University School of Medicine, Hangzhou, 310000 Zhejiang China; 4 Bone Marrow Transplantation Center of the First Affiliated Hospital, Institute of Immunology, Zhejiang University School of Medicine, Hangzhou, 310000 Zhejiang China; 5 Department of Pathology, the Second Affiliated Hospital, Zhejiang University School of Medicine, Hangzhou, 310000 Zhejiang China; 6 Department of General Surgery, Sir Run Run Shaw Hospital, Zhejiang University School of Medicine, Hangzhou, 310000 Zhejiang China

**Keywords:** Gastric cancer, TCGA, Immune-related lncRNA pair, LASSO, Prognostic prediction, Immune response

## Abstract

**Background:**

Gastric cancer (GC), the most commonly diagnosed cancer worldwide with poor 5-year survival rate in advanced stages. Although immune-related and survival-related biomarkers, which typically comprise aberrantly expressed long non-coding RNAs (lncRNAs) and genes, have been identified, there are no reports of immune-related lncRNA pair (IRLP) signatures for GC.

**Methods:**

In this study, we acquired lncRNA expression profiles from The Cancer Genome Atlas (TCGA) and used the least absolute shrinkage and selection operator (LASSO) Cox proportional hazards model (iteration = 1000) to develop a IRLP prognostic signature. The area under curve (AUC) was used to assess the prognosis predictive power. The multivariate Cox regression analysis was performed to identify whether this signature was an independent prognostic factor. The immune cell infiltration analysis was performed between the two risk groups. Last, molecular experiments were performed to explore LINC01082 is involved in the development of GC.

**Results:**

We acquired lncRNA expression profiles and used the LASSO Cox model to develop an 18-IRLP signature with a strong prognostic predictive power. The 5-year AUC values of the training, validation, and overall TCGA datasets were 0.77, 0.86, and 0.80, respectively. The different prognostic outcomes between the high- and low-risk groups were determined using our 18-IRLP signature. Moreover, our 18-IRLP signature was an independent prognostic factor as per the multivariate Cox regression analysis, and showed better prognostic evaluation than the traditional TNM staging system as well as other clinical features. We also found differences in cancer-associated fibroblast and macrophage M2 infiltration and the expression of PD-L1, CTLA4, LAG3, and HLA were also observed between the two risk groups (P < 0.05). Analysis of biological functions revealed that target genes of the lncRNAs in the IRLP signature were enriched in focal adhesion and regulation of actin cytoskeleton. Finally, as one of significant candidates of IRLP signature, overexpression of LINC01082 suppressed the invasion ability of GC cells as well as PD-L1 expression profiles.

**Conclusions:**

Our novel 18-IRLP signature provides new insights regarding immunological biomarkers, imparts a better understanding of the tumor immune microenvironment, and can be used for predicting prognosis and evaluating immune response in GC.

**Supplementary Information:**

The online version contains supplementary material available at 10.1186/s12935-022-02493-2.

## Introduction

Gastric cancer (GC) is the most commonly diagnosed cancer worldwide, and was the most common cause of cancer-related deaths in 2020 [1]. With an estimated 1.09 million new cases and 769,000 deaths, GC has the fifth highest global incidence and fourth highest rate of mortality [1]. In Eastern Asia, GC is the second most common malignancy [2]. In China, GC has the fifth highest rate of morbidity and the third highest rate of mortality in females, and the second highest rate of morbidity and the third highest rate of mortality in males, indicating that GC is a major burden for both the sexes [3]. After conventional therapeutic intervention, the 5-year survival rate of patients with surgically treated stages IA and IB GC is 60–80%. However, in case of patients undergoing surgery for stage III GC, the 5-year survival rate is 18–50% [4]. Thus, there is an urgent need to identify novel tumor prognostic markers for patients with GC.

Tumor cells can functionally shape their microenvironment by secreting various cytokines, chemokines, and other factors [5]. Cancer immunotherapy is widely used for the treatment of human cancers [6]. Over the past few decades, different inhibitory receptors that play an important role in reducing anti-tumor immune responses have been identified. These include cytotoxic T lymphocyte-associated antigen-4 (CTLA4), programmed cell death protein-1 (PD-1), and programmed death-ligand 1 (PD-L1) [7]. Immune checkpoint blockade is being integrated into first-line therapy and early treatment for GC to provide benefits to a larger proportion of patients [8]. Other immune-related biomarkers have also been identified, and these pave the way for more effective immunotherapy [9] and emphasize the potential of immunotherapy as a promising therapeutic approach for GC.

Long non-coding RNAs (lncRNAs) are large RNAs (> 200 nucleotides) with heterogeneous biological functions. LncRNA aberrations have been associated with various human cancers [10]. They contribute considerably toward intercellular communication, revealing the complex interactions among the tumor cells, tumor microenvironment (TME) cells, and immune cells [11]. Therapeutic targeting of lncRNAs is being explored as a promising research topic [12]. Recently, there have been many reports on the involvement of lncRNAs in the occurrence and development of GC.

The survival outcomes of patients can be effectively predicted using prognosis signatures comprising biomarkers, such as aberrantly expressed genes and lncRNAs. Recently, immune-related biomarkers, such as immune-related genes (IRGs) and immune-related lncRNA signatures have been reported for prognosis prediction. For instance, Xu et al. have developed a seven-IRG risk signature (*LCN12*, *CCL21*, *RNASE2*, *CGB5*, *NRG4*, *AGTR1*, and *NPR3*) for predicting overall survival (OS) in male patients with GC [13]. Another study identified an immune-related prognostic signature consisting of ten IRGs (*S100A12*, *DEFB126*, *KAL1*, *APOH*, *CGB5*, *GRP*, *GLP2R*, *LGR6*, *PTGER3*, and *CTLA4*) for GC [14]. Wang et al. used gene expression data from The Cancer Genome Atlas (TCGA) database to develop a prognostic signature with 19 immune-related lncRNAs for GC [15]. However, because of the use of different platforms, it is challenging to compare and validate different datasets. Thus, the concept of gene–lncRNA pairs, which encompasses a comparison between the expression levels of two genes/lncRNAs, has recently emerged. Zhao et al. have identified a signature of 14 immune-related gene pairs (IRGPs) comprising 25 unique genes in GC [16]. An 11-IRGP signature associated with TP53 has also been developed for predicting the OS of patients with GC [17]. However, it is noteworthy that currently there are no reports of immune-related lncRNA pair (IRLP) signatures for GC.

Thus, in the present study, we performed bioinformatics and biological validation analyses to develop a novel 18-IRLP signature, which comprised 27 immune-related lncRNAs, for the prognosis of GC as well as the evaluation of the related immune response. Our IRLP signature will provide a better understanding of the tumor immune microenvironment and the therapeutic response in GC.

## Material and methods

### Acquisition of gene expression and clinical data

RNA-sequencing gene expression data (fragments per kilobase per million files) and clinical information of GC patients were downloaded from TCGA database (https://portal.gdc.cancer.gov/) [18] on April 2021. All files were downloaded using the “TCGAbiolinks” R package [19]. A total of 407 samples were obtained, including 375 cancer and 32 normal samples. We then annotated all mRNAs and lncRNAs using GENCODE reference annotation (https://www.gencodegenes.org/) [20] GTF files (gencode.v37.annotation.gtf and gencode.v37.long_noncoding_RNAs.gtf.gz, respectively). From the GC clinical data, we selected samples with a survival time of more than 30 days and with detailed clinicopathological data. Finally, we selected a total of 303 patients with GC for further study.

### Mining immune-related LncRNAs in GC

To identify immune-related lncRNAs, we used an algorithm developed by Li et al. [21] in 2020, which is a three-step computational framework called “ImmLnc” for identifying lncRNA regulators of immune-related pathways in human cancers. The R package “ImmulancRNA” was used to calculate the tumor purity and partial correlation coefficient, and identify lncRNA–pathway pairs. First, gene and lncRNA expression profiles were collected from patients with the same tumor. Next, the tumor purity of each sample was calculated. All coding genes were ranked based on the correlation of their expression with specific lncRNAs. The rank score (RS) was then calculated for each lncRNA–gene pair. All genes were ranked based on the RS scores for each candidate lncRNA, and then subjected to enrichment analysis. Finally, the activity of each lncRNA in the immune pathway (Additional file 1: Table S1) was calculated based on a differential gene enrichment analysis. The activity of each lncRNA in the immune pathway (lncRES) was calculated based on an improved gene set enrichment analysis. The lncRES scores ranged from -1 to 1. LncRNA–pathway pairs with absolute lncRES scores > 0.995 and false discovery rate (FDR) < 0.05 were considered significant.

### Computation of candidate immune-related LncRNA pairs in GC

To further reduce the number of lncRNAs, we selected differentially expressed immune-related lncRNAs (DEimmunelncRNAs) between the normal (n = 32) and tumor (n = 375) tissues by considering FDR < 0.05 and fold change > 1.5 as the cutoff criteria according to the “limma” R package [22]. Pairwise comparisons were then performed based on lncRNA expression levels in each sample to obtain IRLPs. In one specific sample, if the expression value of the first lncRNA was greater than that of the second lncRNA, the score of the IRLP in that sample was considered to be 1; otherwise, it was considered to be 0. That is,

if Expr_*lncRNA1*_ > Expr_*lncRNA2*_, then IRLPs = 1, else, IRLPs = 0.

Here, Expr_*lncRNA1*_ is the expression value of the first immune-related lncRNA, and Expr_*lncRNA2*_ is the expression value of the second immune-related lncRNA. After calculating the score of each IRLP in each GC sample, we removed IRLPs with low variation (IRLPs with a score of 1 or 0 in less than 20% of all samples). The remaining IRLPs were subsequently selected as the first candidate IRLPs for conducting further analyses. Finally, univariate Cox regression analysis was used and P-value < 0.01 was considered significant for selecting prognosis-related IRLPs.

### Construction of a prognostic IRLP signature using a LASSO model

All 303 patients with GC from TCGA dataset were randomly categorized into two sets in a 7:3 ratio in the training set (n = 212) and validation set (n = 91) to avoid the influence of random assignment bias. The training and validation sets showed no significant differences in the distribution of age, sex, tumor stage, grade, pathological tumor (pT), node (pN), metastasis (pM) stages, and patient survival status (Table 1). We used the Cox proportional hazards model (iteration = 1,000) with a least absolute shrinkage and selection operator (LASSO) penalty on the abovementioned prognostic-related IRLPs to determine the optimal gene model using the “glmnet” R package. After 1,000 iterations, gene groups were obtained, and the most frequently occurring gene combinations were considered as the final IRLP signature for GC.

**Table 1:** Clinical characteristics of GC patients in the training and validation set

Clinical features	Level	Overall	Training	Validation	Statistical P-value
Samples		303	212	91	
Survival status (%)	Alive	177 (58.4)	121 (57.1)	56 (61.5)	0.552
	Dead	126 (41.6)	91 (42.9)	35 (38.5)	
Age (%)	< 65	134 (44.2)	97 (45.8)	37 (40.7)	0.489
	> = 65	169 (55.8)	115 (54.2)	54 (59.3)	
Sex (%)	Female	112 (37.0)	75 (35.4)	37 (40.7)	0.457
	Male	191 (63.0)	137 (64.6)	54 (59.3)	
pStage (%)	I/II	138 (45.5)	92 (43.4)	46 (50.5)	0.308
	III/IV	165 (54.5)	120 (56.6)	45 (49.5)	
pT (%)	T1	12 (4.0)	11 (5.2)	1 (1.1)	0.265
	T2	63 (20.8)	42 (19.8)	21 (23.1)	
	T3	148 (48.8)	100 (47.2)	48 (52.7)	
	T4	80 (26.4)	59 (27.8)	21 (23.1)	
pN (%)	N0	93 (30.7)	67 (31.6)	26 (28.6)	0.566
	N1	81 (26.7)	57 (26.9)	24 (26.4)	
	N2	65 (21.5)	41 (19.3)	24 (26.4)	
	N3	64 (21.1)	47 (22.2)	17 (18.7)	
pM (%)	M0	282 (93.1)	197 (92.9)	85 (93.4)	1
	M1	21 (6.9)	15 (7.1)	6 (6.6)	
Grade (differentiated, %)	Well	7 (2.3)	7 (3.3)	0 (0.0)	0.19
	Moderately	100 (33.0)	74 (34.9)	26 (28.6)	
	Poorly	188 (62.0)	126 (59.4)	62 (68.1)	
	Undifferentiated	8 (2.6)	5 (2.4)	3 (3.3)	

^*^ The statistical differences between two groups were tested by χ^2^ or Fisher exact tests, if appropriate. pT: pathological T stage. pN: pathological N stage. pM: pathological M stage

### Prognostic evaluation and clinical association of the IRLP signature in GC

Based on the above IRGP signature and its regression coefficient, the risk score of each patient with GC was calculated as follows:

$$Risk \, score=\Sigma \, IRLPs*coefficient$$

Here, IRLPs refer to the immune-related lncRNA pairs and the coefficient refers to the regression coefficient. Accordingly, all samples were categorized into low-risk and high-risk groups based on the median risk score. Kaplan–Meier (K-M) survival curves and log-rank tests were used to evaluate the differences in OS between the low-risk and high-risk groups. Time-dependent receiver operating characteristic (ROC) curve analysis was used to evaluate the sensitivity and specificity of the IRLPs. The “KMsurv” R package was used to generate the K-M plot of the two risk groups and the “survivalROC” package was used to evaluate the ROC curve of the model and calculate the area under the ROC curve (AUC).

Multivariate Cox regression analysis was used to investigate whether the IRLP signature could be an independent prognostic factor for GC. The ROC curve was used to compare our IRLP signature with the traditional TNM staging system for prognostic prediction. The “CancerSubtypes” R package [23] was used to determine the molecular subtypes of all GC samples using the consensus clustering method. The package CancerSubtypes integrates the current common biology methods for cancer subtypes identification and provides a standardized framework for cancer subtype analysis based multi-omics data, such as gene expression. There are four main computational methods: Consensus clustering (CC), Consensus non-negative matrix factorization (CNMF), Integrative clustering (iCluster), and Similarity network fusion (SNF), and a combined method named as SNF-CC to combine SNF and CC together. A nomogram was used to display the combination of clinical characteristics with the IRLP signature using the “rms” and the “regplot” R packages. We used the concordance index (C-index) to evaluate the discriminative power of the nomogram, and drew a calibration curve to evaluate the accuracy of the prediction. Purpose of the calibration plot is to visually see whether the model used to produce predicted probabilities is reflective of the true probability of the sample. The closer the dots are to the 45 degree line, the better the model. To better display the distribution of the clinical features between the high- and low-risk groups, we used the “ComplexHeatmap” R package.

### Estimation of immune cell infiltration and immune-related activities

We performed immune infiltration estimation using TIMER 2.0 (http://timer.comp-genomics.org/), which is used for immune infiltration across diverse cancer types. The estimation results included expression profiles provided by immunedeconv [24] using TIMER [25], CIBERSORT [26], quanTIseq [27], xCell [28], MCP-counter [29], and EPIC algorithms [30]. R package “ggplot2” was used to show the coefficients of the relationship between immune cell infiltration and patient risk scores. The “ggpubr” and “ggplot2” packages were used to display the differences in distribution between the risk groups (low-risk and high-risk groups) and immune cell infiltration. The gene expression of immune checkpoint inhibitors (ICIs), including programmed cell death 1 (PCDC1, also known as PD-1), CD274 (also known as PD-L1), cytotoxic T-lymphocyte associated protein 4 (CTLA4), and lymphocyte-activation gene 3 (LAG3), was evaluated in the high- and low-risk groups. We also explored the distribution of the expression of the human leukocyte antigen (HLA) family in the two risk groups.

### Biological functions of the IRLP signature in GC

To investigate the association between targeted drugs and our IRLP signature for the treatment of patients with GC, we used the “pRRophetic” R package, which is an R package used for predicting clinical chemotherapeutic response based on tumor gene expression levels [31]. The package can be used to estimate the chemotherapeutic response determined by the half-maximal inhibitory concentration (IC50) of each patient with GC. To analyze the lncRNA target genes, we used a correlation coefficient > 0.6 and a P-value < 0.05 to identify potential target genes. The “clusterProfiler” R package was used to perform pathway enrichment analysis of these target genes. FDR < 0.05 were considered significant. Additionally, the K-M survival plot was used to determine the prognosis and lncRNA expression profiles of the patients. A violin plot was used to show the differences in the expression of all signature-related lncRNAs between the tumor and normal tissues. Finally, the correlation coefficient between two lncRNAs was calculated.

### Cell culture and transfection

The human GC cell line HGC-27 was purchased from the Procell Life Science&Technology Co., Ltd (Wuhan, China). The cells were cultured in RPMI-1640 medium (#PM150110, Procell) with 20% fetal bovine serum (#164210-500, Procell) and 1% penicillin–streptomycin (#PB180120, Procell) in an environment with 5% CO_2_ at 37 °C. The cells were seeded in 6-well culture plates, and cell transfection was performed when the cell confluence reached 60–70%. For LINC01082 overexpression, the pcDNA3.1-LINC01082 plasmid was obtained from GENEWIZ (Suzhou, China). The negative control group of empty vector was referred to as the NC group. For LINC01082 knock down, the si-LINC01082 and the corresponding negative control were synthesized from Ribobio (Guangzhou, China). The cells were transfected with 2.5 µg plasmid DNA and 4 µl Lipo8000™ (Beyotime, China) per well following the manufacturer’s protocol. All cells were collected 48 h after transfection.

### RNA extraction and quantitative real-time PCR analysis

Total RNA was extracted using TRIzol™ Reagent (Thermo Fisher Scientific, China) and reverse-transcribed into complementary DNA (cDNA) using the All-In-One 5X RT MasterMix Kit (abmgood, Canada) by following the manufacturer’s protocol. Quantitative real-time polymerase chain reaction (qRT-PCR) was performed using the 2 × Taq PCR Master Mix (abmgood, Canada). The protocol of each qRT-PCR cycle was: 94 °C for 10 min, 94 °C for 30 s, 60 °C for 30 s, and 72 °C for 60 s, which was repeated 40 times. The forward primer sequence of LINC01082 was 5ʹ-CGGACTCTATCGAGGCACAC-3ʹ and the reverse primer sequence was 5ʹ-GCTGCTCTCGAGTTCCCTAC-3ʹ. The forward primer sequence of GAPDH was 5ʹ-CAAATTCCATGGCACCGTCA-3ʹ and the reverse primer sequence was 5ʹ-GACTCCACGACGTACTCAGC-3ʹ.

### Transwell assays

Transwell migration assays were performed to determine the migration potential of the transfected cells using Transwell devices containing microporous 8-μm membranes (Becton,Dickinson and Company, USA) in 24-well plates. First, 500 μL Dulbecco’s modified Eagle’s medium (DMEM) containing 20% FBS to each well was added into 24-well plates. Next, approximately 4 × 10^4^ transfected cells were resuspended in 100 μL DMEM seeded in the chamber. The cells were cultured for 48 h at 37 °C with 5% CO_2_. Then, 1 mL of 4% formaldehyde solution was added to each well and fixed at room temperature for 10 min. Then, the wells were washed with 1 × phosphate-buffered saline (PBS), and 1 mL of 0.1% crystal violet solution (Solarbio, Beijing, China) was added to each well. After staining for 30 min, the wells were washed three times with 1 × PBS and dried. Finally, all non-migrated cells were scraped using a cotton bud. Cells were counted in five randomly and uniformly selected visual fields of the membrane.

### Cell proliferation assays

A total of 1 × 10^3^ transfected cells were seeded in the 96-well plates for 0, 24, 48, 72 and 96 h. The cell counting kit 8 (CCK-8, #ab228554, Abcam) was selected, and 10 μL of CCK-8 was added per well. After being incubated for 1 h at 37 °C, we detected the absorbance of each well at a wavelength of 450 nm. Individual assays were performed for three times.

### Western blotting

Total proteins were extracted by the Cell Lysis Buffer (Beyotime, China). The protein quantification from the total extracted cell protein lysates was detected by the BCA protein assay, then the same 10 μg of protein samples were separated by the SDS-PAGE and transferred onto PVDF membranes. The proteins on the membrane were then blocked with 5% skim milk for 30 min and incubated overnight at 4 °C with anti-E-Cadherin (#20874-1-AP, Proteintech, China, 1:5000), anti-MMP2 (#10373-2-AP, Proteintech, China, 1:500), anti-ZO-1 (#13663, Cell Signaling Technology, USA, 1:1000), and anti-GAPDH (#10494-1-AP, Proteintech, China, 1:10,000) antibodies. The membranes were incubated with the corresponding secondary antibody (#SA00001-1, #SA00001-2, Proteintech, China, 1:5000) at room temperature for 30 min. Last, membranes were incubated with chemiluminescent substrate according to the manufacture’s protocol (#E412, Vazyme, China) and visualized using Tanon-4600 automatic chemiluminescence/fluorescence image analysis system (Shanghai, China). The experiments were repeated twice.

### Statistical analysis

All statistical analyses were conducted using the latest version of the R programming language (version 4.1.0). Mann–Whitney–Wilcoxon test was used to compare two independent non-parametric samples. Univariate and multivariate Cox proportional hazards regression models were used to identify the prognostic factors and independent prognostic factors, respectively. Spearman’s correlation coefficient was used to assess the correlation between two factors. Relative RNA expression levels of LINC01082 in overexpressed groups (OE) compared with negative control groups (NC) were calculated using 2^−ΔΔCT^, we performed the following four steps: (1) The mean CT value of each gene was calculated for each group; (2) ΔCT values: ΔCT is the difference between the target cDNA and endogenous reference in the CT value (GAPDH; ΔCT = CT _lncRNA_ – CT _GAPDH_); (3) ΔΔCT values: –ΔΔCT = –(ΔCT _OE_ – ΔCT _NC_); and (4) 2^−ΔΔCT^ values: After 2^−ΔΔCT^ calculation, the relative expression of the target gene between OE and NC was finally obtained. The calculation methods of LINC01082-si and NC groups were the same as above. All reported P-values were two-tailed, and P-values < 0.05, were considered significant.

## Results

### Identification of candidate immune-related LncRNAs in GC

**Fig. 1:**
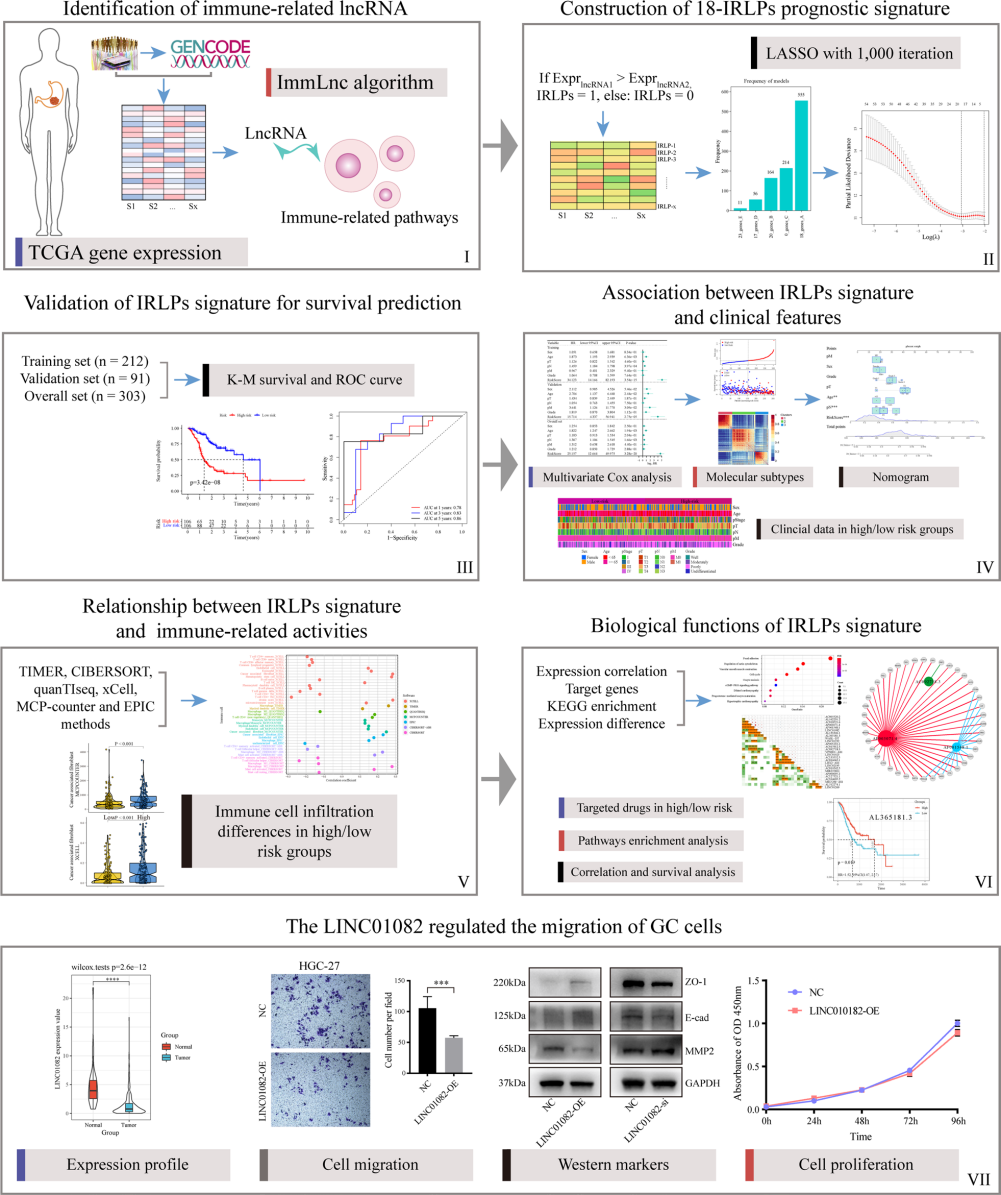
Flow chart of the present study. A novel immune-related lncRNA pair (IRLP) signature for predicting prognosis and evaluating immune response in gastric cancer (GC) was obtained via a bioinformatics and biological validation-based study

**Fig. 2:**
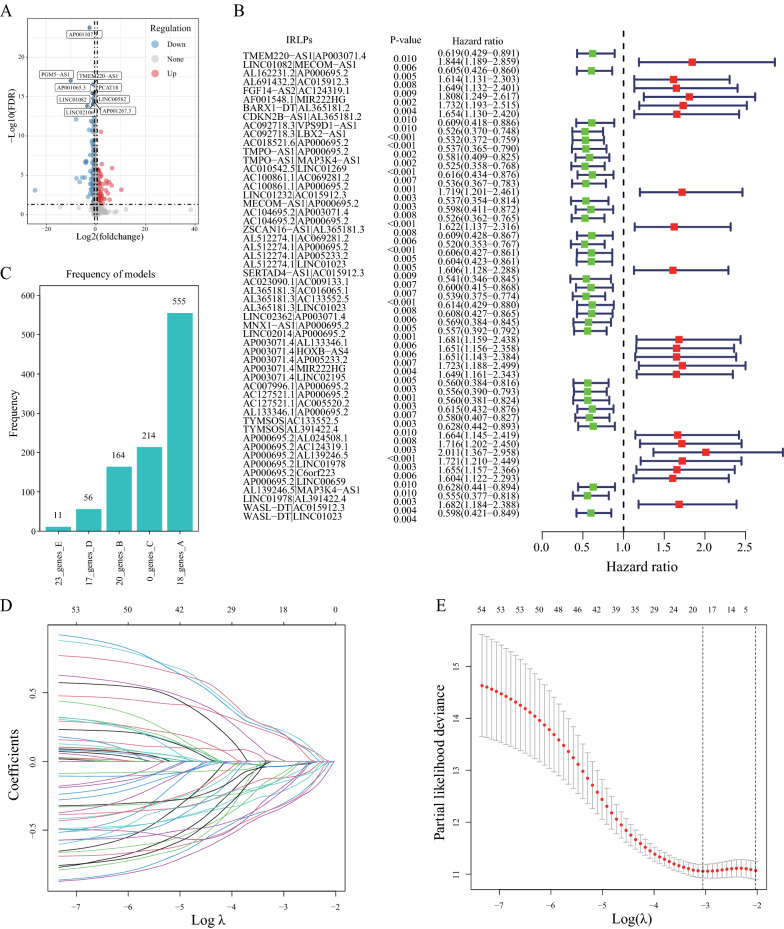
Identification and construction of the immune-related lncRNA pair (IRLP) signature in gastric cancer (GC). **A** The volcano plot of differentially expressed immune-related lncRNAs (DEimmunelncRNAs) between the tumor and normal tissues from The Cancer Genome Atlas (TCGA) data. **B** The forest plot of prognostic-related IRLPs as per the univariate Cox proportional hazards regression model. **C** The frequencies of all gene model occurrences with more than 1000 cycles. **D** Least absolute shrinkage and selection operator (LASSO) coefficient profile plots generated against log (lambda) value. **E** Selection of the optimal parameter (lambda) in the LASSO model for GC

We performed a multi-step integrated analysis to construct an IRLP signature for predicting prognosis and evaluating immunotherapeutic response in GC (Fig. 1). We used the gene expression profiles from TCGA database and the computational framework “ImmLnc” to identify 1704 immune-related candidate lncRNAs for determining lncRNA regulators of the immune-related pathways in GC. We further screened the lncRNAs to avoid very low expression lncRNAs, and obtained 1448 lncRNAs with expression values greater than 0 in 70% of all samples. Using the “limma” R package, a total of 162 DEimmunelncRNAs between the tumor and normal samples were selected, of which 74 were upregulated and 88 were downregulated (Fig. 2A).

### Construction of an 18-IRLP prognostic signature in GC

Based on above 162 DEimmunelncRNAs that were selected, we performed pairwise comparisons using the lncRNA expression levels in each sample to obtain IRLPs. After eliminating IRLPs that did not meet the inclusion criteria, we retained 6677 IRLPs for subsequent analyses. Using the univariate Cox proportional hazards regression model, a total of 54 prognosis-related IRLPs were screened (Fig. 2B, Additional file 2: Table S2). To avoid the influence of random assignment bias, all 303 patients were categorized into training and validation sets, which showed no significant differences in the distribution of the clinical features. The training and validation sets showed no significant differences in the distribution of the clinical features (Table 1). To construct a stable prognostic evaluation model, the 54 selected IRLPs underwent Cox proportional hazards regression with tenfold cross-validation to generate the most optimal gene signature. We performed 1000 iterations and included five gene groups for further analyses. As shown in Fig. 2C, an 18-IRLP gene signature, including 27 immune-related lncRNAs, was selected. The selected model had the highest frequency of 555 times higher than that of all other models (Table 2). Therefore, this 18-IRLP gene model was considered to be the most suitable for generating the immune signature in GC (Fig. 2D, E).

**Table 2:** Prognostic-related 18-IRLPs signature by univariate Cox and LASSO regression analysis

IRLPs	LncRNA1	LncRNA2	LASSO Coefficient	Univariate Cox HR	Univariate Cox P-value
LINC01082|MECOM-AS1	LINC01082	MECOM-AS1	0.182461199	1.844162	0.006239
AL162231.2|AP000695.2	AL162231.2	AP000695.2	− 0.090117905	0.604829	0.005055
AF001548.1|MIR222HG	AF001548.1	MIR222HG	0.013909284	1.807629	0.001712
AC092718.3|VPS9D1-AS1	AC092718.3	VPS9D1-AS1	− 0.1216813	0.6087	0.009608
AC092718.3|LBX2-AS1	AC092718.3	LBX2-AS1	− 0.315792113	0.525771	0.000353
AC018521.6|AP000695.2	AC018521.6	AP000695.2	− 0.221816573	0.531631	0.000512
AC010542.5|LINC01269	AC010542.5	LINC01269	− 0.322756413	0.524657	0.000923
LINC01232|AC015912.3	LINC01232	AC015912.3	0.227198005	1.719174	0.003078
AC104695.2|AP000695.2	AC104695.2	AP000695.2	− 0.120394541	0.525991	0.000775
AL512274.1|AP005233.2	AL512274.1	AP005233.2	− 0.223782758	0.606419	0.005118
AL512274.1|LINC01023	AL512274.1	LINC01023	− 0.035507173	0.603742	0.005325
AL365181.3|AC016065.1	AL365181.3	AC016065.1	− 0.036905121	0.600231	0.006761
AL365181.3|AC133552.5	AL365181.3	AC133552.5	− 0.13115007	0.538969	0.000808
AP003071.4|AL133346.1	AP003071.4	AL133346.1	0.20335131	1.681189	0.006167
AP003071.4|LINC02195	AP003071.4	LINC02195	0.210730199	1.649251	0.005243
AC127521.1|AC005520.2	AC127521.1	AC005520.2	− 0.153210626	0.560476	0.003205
AL133346.1|AP000695.2	AL133346.1	AP000695.2	− 0.196021258	0.615435	0.007012
WASL-DT|LINC01023	WASL-DT	LINC01023	− 0.340532934	0.597772	0.004098

*IRLPs* immune-related lncRNA pairs, *HR* hazard ratio

### Evaluation of survival prediction and validation of the IRLP signature

**Fig. 3:**
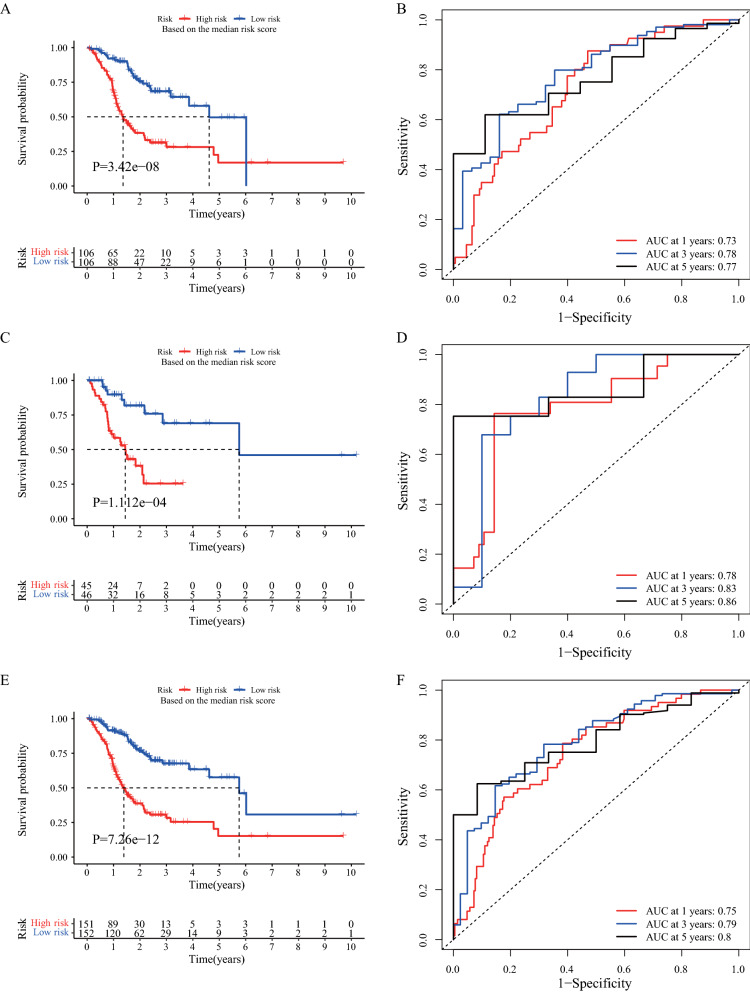
The prognostic evaluation and validation of the immune-related lncRNA pair (IRLP) signature in gastric cancer (GC). **A** The Kaplan–Meier (K-M) plot between the high- and low-risk groups in the training set (n = 212). **B** The time-dependent receiver operating characteristic (ROC) curves for 1-, 3-, and 5-years in the training set. **C** The K-M plot between the high- and low-risk groups in the validation set (n = 91). **D** The time-dependent ROC curves for 1-, 3-, and 5-years in the validation set. **E** The K-M plot between the high- and low-risk groups in The Cancer Genome Atlas (TCGA) set (n = 303). **F** The time-dependent ROC curves for 1-, 3-, and 5-years in TCGA set

We used the 18-IRLP signature and calculated the risk score for each patient in the training set to construct a prognostic risk model for patients with GC. Based on the median risk score, we found that the high-risk group exhibited poorer prognosis than the low-risk group in the training, validation, and TCGA sets (log-rank test P-value < 0.05; Fig. 3A, C, and E). The 1-, 3-, and 5-year AUC values of the training set were 0.73, 0.78, and 0.77, respectively (Fig. 3B). In addition, the AUC values in the validation and TCGA sets were greater than 0.75 in case of 1-, 3-, and 5-year AUC (Fig. 3D, F). These results suggest that our 18-IRLPs signature is a strong prognostic predictor for GC.

### Association between the IRLP signature and clinical features

**Fig. 4:**
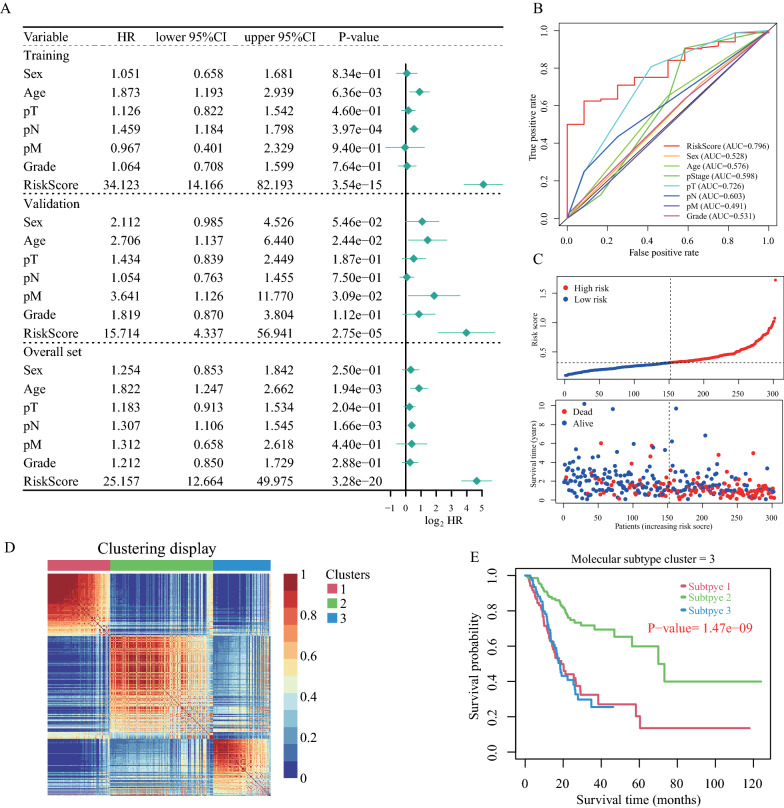
The association between immune-related lncRNA pair (IRLP) signature and clinical features. **A** The 18-IRLP signature can be considered an independent prognostic factor for gastric cancer (GC) based on the results of the multivariate Cox regression analysis. **B** The 5-year time-dependent receiver operating characteristic (ROC) curves of the 18-IRLPs signature, traditional TNM staging, and other clinical features. **C** The distribution of the risk score and survival status in the patients. **D** The results of molecular subtype clustering using the consensus clustering method. **E** The Kaplan–Meier (K-M) plot of the three subtype clusters (log-rank test, P-value < 0.001)

To explore the association between the IRLP signature and clinical features, along with other clinical factors, including sex, age, pT stage, pN stage, pM stage, and grade, the multivariate Cox regression analysis revealed that our 18-IRLP signature was an independent prognostic factor for GC (Fig. 4A). As shown in Fig. 4B, we determined that our model was better than the traditional TNM staging system, as well as other clinical features in terms of prognostic evaluation in GC. Figure 4C shows the distribution of the risk score and survival status.

To explore whether our IRLP signature was useful in determining the molecular clusters for GC, we used a consensus clustering method to stratify all the samples, and identified three molecular subtype clusters: subtype 1, subtype 2, and subtype 3 (Fig. 4D). We found significant differences in the survival rates among the patients in the three subtype clusters, especially in case of subtype 2 (log-rank test, P < 0.001, Fig. 4E). These results suggest that our IRLP signature can be used for molecular subtyping in further studies.

**Fig. 5:**
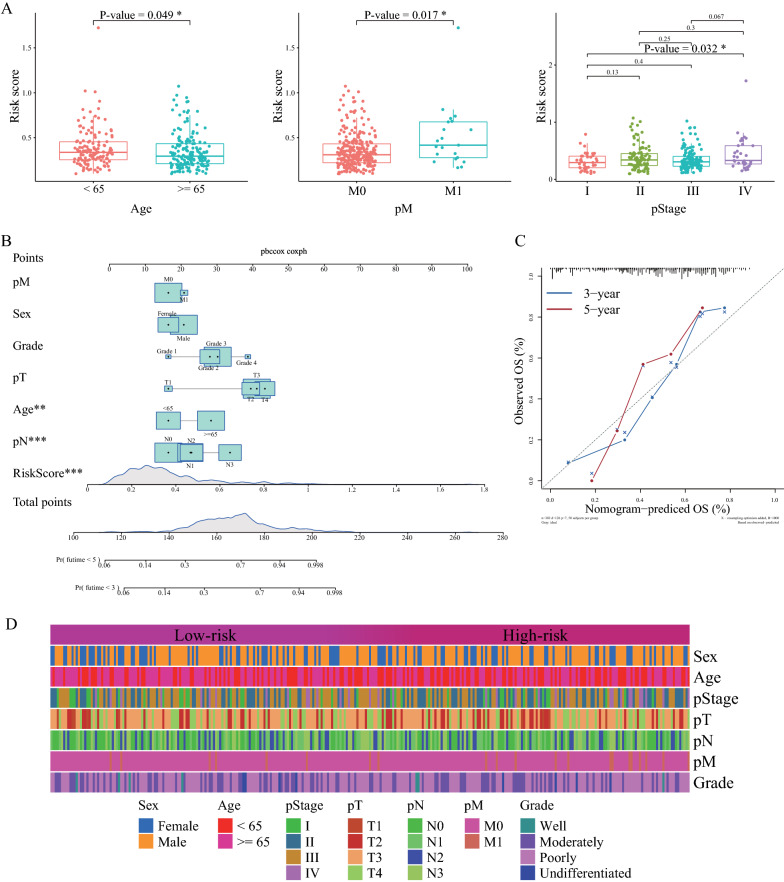
The differences in the distribution of risk score according to clinicopathological features and construction of a nomogram for immune-related lncRNA pair (IRLP) signature. **A** The differences in the risk score based on age, pM, and pStage. **B** The nomogram for prognostic prediction in gastric cancer (GC). **C** The calibration curve for evaluating the prediction accuracy of the model. **D** The heatmap of the distribution of clinical features between the high-risk and low-risk groups

We then examined the differences in the risk score according to different clinicopathological features. The distribution of the risk scores was significantly different with respect to age, pM stage, and pathological stage I with IV (P < 0.05, Fig. 5A). Moreover, we integrated the abovementioned clinicopathological indicators with sex, age, pT stage, pN stage, pM stage, grade, and our 18-IRLPs signature. We constructed a nomogram to further improve the accuracy of the prognostic prediction model for GC (Fig. 5B). The C-index of this nomogram was 0.762, which indicates a strong prognostic prediction ability for GC. The calibration curve used to evaluate the prediction accuracy of the model is shown in Fig. 5C. To better display the distribution of the clinical information between the high-risk and low-risk groups, a heatmap of the distribution of clinical features is illustrated in Fig. 5D.

### Relationship between the IRLP signature and immune-related activities

**Fig. 6:**
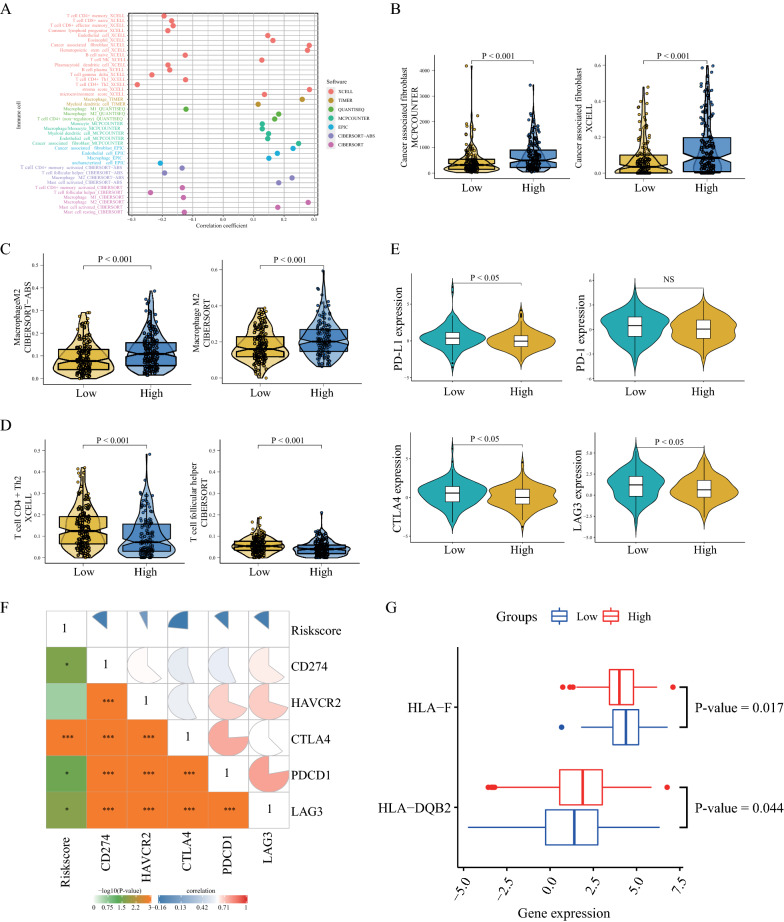
The relationship between immune-related lncRNA pair (IRLP) signature and immune-related activities. **A** The bubble plot of immune cell infiltration and risk scores. **B**–**D** The differences in distribution of immune cell infiltration between the high- and low-risk groups. Cancer-associated fibroblasts (**B**), M2 macrophages (**C**), and T cell-associated activities (**D**). **E** The distribution of expression of immune checkpoint inhibitors (ICIs) between the high- and low-risk groups. NS indicates insignificant values. **F** The correlation coefficients of the risk score and ICIs. **G** The box plot of HLA-F and HLA-DQB2 expression between the two risk groups

Immune cell infiltration is associated with cancer prognosis [32]. We explored the differences in immune cell infiltration between the high- and low-risk groups. We first estimated the immune infiltration status in GC by using TIMER, CIBERSORT, quanTIseq, xCell, MCP-counter, and EPIC algorithms. As shown in Fig. 6A, we determined that several immune-related responses, such as those associated with B cells, CD4 + T cells, CD8 + T cells, follicular helper T cells, and M1 macrophages had a negative correlation with the risk scores (correlation coefficient < 0). However, M2 macrophages, cancer-associated fibroblasts, and endothelial cells were positively correlated with the patient risk scores (correlation coefficient > 0). Additional file 3: Figure S1 shows a heatmap of immune cell infiltration estimated using all above mentioned methods. Moreover, cancer-associated fibroblasts (Fig. 6B), M2 macrophages (Fig. 6C), and T cell-associated infiltration (Fig. 6D) showed differential infiltration (P-value < 0.001) between the high- and low-risk groups.

We next explored the correlation between the risk score and ICI-related biomarkers. Significant differences were observed in the expression of PD-L1, CTLA4, and LAG3 between the high- and low-risk groups (P < 0.05); however, the difference in PD-1 expression was not significant (Fig. 6E). The correlation coefficients that were computed to determine an association between the 18-IRLP signature and the ICIs (Fig. 6F) suggested a weak correlation.

As shown in Fig. 6G, the expression of HLA-F was downregulated in the high-risk group, whereas, the expression of HLA-DQB2 was upregulated in the high-risk group, indicating that this possibly led to tumor immune evasion (P-value < 0.05).

### Biological functions of the IRLP signature in GC

Considering the importance of targeted drugs in the treatment of patients with GC, we determined the drugs that were more sensitive in the high- and low-risk groups based on the IC50 value (Additional file 4: Figure S2). According to the IC50 values, we found that bryostatin-1, dasatinib, KU-55933, MG-132, pazopanib, and temsirolimus were more effective in the high-risk patients.

**Fig. 7:**
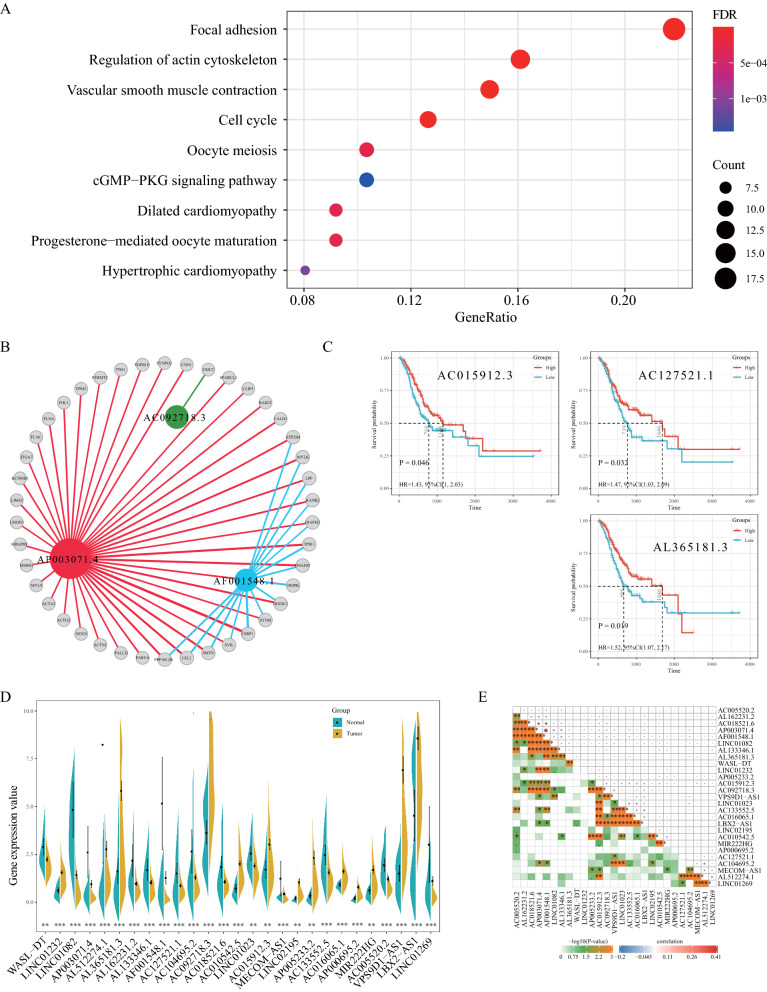
Biological functions of the immune-related lncRNA pair (IRLP) signature in gastric cancer (GC). **A** Kyoto Encyclopedia of Genes and Genomes (KEGG) pathway enrichment analysis of the target genes of the lncRNAs in our signature. **B** Target gene regulatory network with correlation coefficient greater than 0.7. **C** The Kaplan–Meier (K-M) survival plot of three prognostic-related lncRNAs.**D** Distribution of lncRNA expression in our signature between the tumor and normal samples. * P-value < 0.05; ** P-value < 0.01; *** P-value < 0.001; ns: P-value ≥ 0.05. **E** Correlation analysis of lncRNA expression with each other

To explore the biological functions of the 18-IRLP signature, we identified target genes of the lncRNAs. By considering correlation coefficient > 0.6, we obtained 190 potential target genes, including 143 positively correlated and 47 negatively correlated genes (Additional file 5: Table S3). The target genes were enriched in several important biological pathways, such as “focal adhesion,” “regulation of actin cytoskeleton,” and “cGMP-PKG signaling pathway” (Fig. 7A). We then screened for target genes with a correlation coefficient > 0.7, and found three significant lncRNAs, namely, AP003071.4, AF001548.1, and AC092718.3 (Fig. 7B). Based on the expression levels, the survival analysis revealed that only three lncRNAs (AC015912.3, AC127521.1, and AL365181.3) were associated with GC prognosis (Fig. 7C) and were differentially expressed in the tumor and normal tissues (Fig. 7D). AP003071.4 and AF001548.1 expression showed a strong positive correlation (correlation coefficient = 0.90; Fig. 7E).

### The LINC01082 regulated the migration of GC cells

**Fig. 8:**
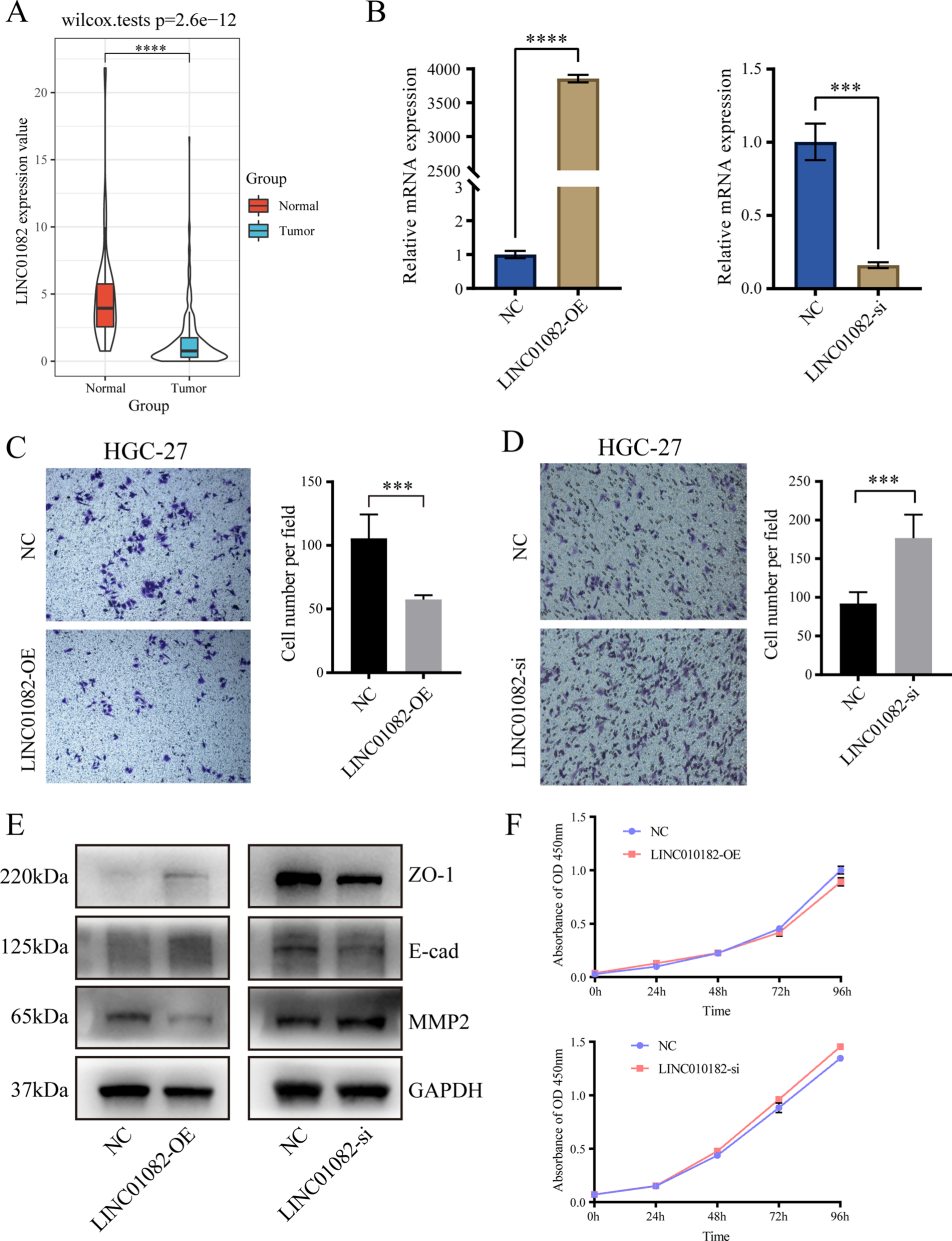
The LINC01082 regulated the migration of gastric cancer (GC) cells. **A** LINC01082 was significantly downregulated in the cancer tissues than in the normal tissues. **B** The overexpression and knock down efficiencies of LINC01082 in HGC-27 cells. **C** Transwell migration assay for overexpression of LINC01082. Original magnification × 20. Scale bar = 200 μm. **D** Transwell migration assay for knock down of LINC01082. Original magnification × 20. Scale bar = 200 μm. **E** The western blotting results of ZO-1, E-cad, and migration relative marker MMP2. **F** Cell proliferation assessment of LINC01082 overexpressing and knock down group *vs* control group by cell counting kit 8 (CCK8) assay. * P-value < 0.05; ** P-value < 0.01; *** P-value < 0.001; **** P-value < 0.0001. NC: negative control group, LINC01082-OE: LINC01082 overexpression group, LINC01082-si: LINC01082 knock down group

To explore the biological behavior of the selected lncRNAs, we performed a molecular validation via functional experiments. Among the many candidates in our signature, we selected an interesting lncRNA named as LINC01082, which has almost never been reported. Based on TCGA data, we found that LINC01082 was significantly downregulated in cancer tissues (Fig. 8A). At the same time, this lncRNA has not been reported in GC, so its biological behavior is worthy of our in-depth study. Results of qRT-PCR displayed substantially increased expression of LINC01082 in pcDNA3.1-LINC01082 overexpressed group (LINC01082-OE) in HGC-27 cells. Decreased expression of LINC01082 was seen in knock down-group (LINC01082-si) compared with NC group (Fig. 8B). Cell migration assay was performed to investigate the regulatory role of LINC01082 in GC cell migration. We demonstrated that it can inhibit cell migration in the LINC01082-OE group than in the control group (Fig. 8C, P-value < 0.001). However, down-regulation of LINC01082 can improve GC cell migration (Fig. 8D, P-value < 0.001). Based on the western blotting results, we also found the expression of tight junction protein 1 (ZO-1, one of Epithelial-Mesenchymal Transition (EMT) relative marker) and E-Cadherin (E-cad, one of EMT relative marker) were increased and matrix metallopeptidase 2 (MMP2, one of migration relative marker) was suppressed by upregulation of LINC01082 (Fig. 8E). However, we found that there was no statistical difference in cell proliferation in the two groups (Fig. 8F). These results suggested that LINC01082 is involved in the migration of GC cells.

## Discussion

In this study, we developed a novel 18-IRLP signature for predicting prognosis and evaluating immune response using a series of bioinformatics and biological validation analyses for GC. Recently, the identification of immune-related biomarkers has paved the way for more effective immunotherapy in human cancers such as GC. Results of preclinical and clinical trials have expanded our understanding of targeted therapy and immunotherapy [9]. Immunotherapy, such as new immune checkpoint regulators, is considered an innovative approach for GC treatment [33]. Several potential immunotherapy target agents, including anti-CTLA4, anti-PD-1/PD-L1, cancer vaccines, and adoptive cell therapy, have become prominent in immunotherapy-based clinical trials for GC.

With the continuous improvement of RNA-sequencing methods, TCGA database, which comprises molecular features of more than 20,000 primary cancers and matched normal samples of 33 cancer types, is increasingly being used in research. Detailed patient prognosis information in TCGA has allowed for an increasing number of studies on prognostic evaluation signatures, including immune-related gene based or lncRNA based prognostic signatures. For example, eight immune-related lncRNAs (LINC00461, LINC01055, ELFN1-AS1, LMO7-AS1, CYP4A22-AS1, AC079612.1, LINC01351, and MIR31HG) related to the prognosis of patients with colorectal cancer have been identified from TCGA database [34]. Zhang et al. [35] have constructed a four immune-related lncRNA prognostic signature for lung cancer based on a series of bioinformatics analyses. In another study, Zhang et al. used univariate and multivariate Cox regression analyses as well as LASSO analysis to build a six immune-related lncRNA signature for bladder cancer [36]. Thus, studies based on gene expression profiles and patient survival data deepen our understanding of the factors affecting human cancer prognosis.

However, because of different sequencing platforms and batch effects, prognostic evaluation models, such as those mentioned above, can be effectively established but cannot be verified by other samples or other data sets. The concept of immune gene pairs solves this problem. A major advantage of this concept is that it is produced by pairwise comparisons and is completely based on gene expression in the tumor of the same patient. Thus, it can overcome the batch effects of different platforms and does not require data standardization. Zhao et al. [16] identified a signature of 14 immune-related gene pairs consisting of 25 unique genes to predict the OS of patients with GC. In another study, a 14-IRGP signature was developed as a novel prognostic marker for predicting survival of patients with head and neck squamous cell carcinoma [37]. As for IRLP signature, one study identified a 21-IRLP signature to predict the clinical outcomes and immunotherapeutic responses in case of head and neck squamous cell carcinoma [38]. However, this is the only relevant study about IRLP signature in tumor so far. Therefore, the construction of an IRLP signature in GC has novel research significance and value.

In this study, we used the R package "ImmulancRNA" was used to calculate the tumor purity and partial correlation coefficient, and identify lncRNA- pathway pairs. It is worth noting that a tumor purity step is very required. Malignant tumor tissue includes not only tumor cells, but also tumor-related normal epithelial cells and stromal cells, immune cells and vascular cells. This is the composition of the tumor's immune microenvironment, and the tumor's percentage is tumor purity. Moreover, infiltrating stromal cells and immune cells are the main components of normal cells in tumor tissue. They not only interfere with tumor signal, but also play an important role in tumor biology [39]. Several studies suggested that genes, whose expression is negatively correlated with tumor purity and positively correlated with immune cell infiltration, are likely to play important roles in immunology [25, 40]. For example, the immune status of glioma patients with different purity was different. The immunophenotype of glioma with low purity was stronger than that of glioma with high purity [41]. Thus, a tumor purity step is required in computation.

Here, if the expression value of the first lncRNA was greater than that of the second lncRNA, the score of the IRLP in that sample was considered to be 1; otherwise, it was considered to be 0. Because the immune gene pairs only need to consider the value of gene expression within one specific sample, we do not need to consider the expression value between different samples, which saves us the problem of inter-sample batch correction. If we need to do more than one dataset validation, it can help us solve the problem of batch correction. Some IRLPs may be specified as constant values (0 or 1) in the dataset for the following reasons [42]: (1) bias caused by a particular platform; (2) biologically preferred transcription characteristics, this does not distinguish the survival of one patient from that of another. No relationship was considered between pairs and prognosis if the expression quantity of lncRNA pairs was 0 or 1 because pairs without a certain rank could not properly predict patient survival outcome [43]. In brief, IRLPs with the same score (0 or 1) was considered uninformative in more than 80% of the samples and removed from the analysis. The biggest advantage of our study is the use of a repeated LASSO (iteration = 1000) method, which is different from traditional LASSO. In the traditional LASSO algorithm, a specific set of genes appears every time the regression is run. However, in our study, we performed a repeated LASSO to generate gene groups after 1,000 iterations. Thus, the gene signature generated was strong and robust. We also compared the performance of our IRLP signature with other reported immune-related genes/lncRNA models in GC. As shown in Additional file 6: Table S4, we found that the 5-year AUC of our IRLP signature (AUC = 0.77) was greater than that in other studies. In addition, the AUC in the validation set and TCGA sets was more than 0.75 for 1-, 3-, and 5-year AUC. Most importantly, this is the first study to report an IRLP-based prognostic evaluation model for GC.

Tumor immunity is a very complex process, which involves many factors. Malignant tumor tissue includes not only tumor cells, but also tumor-related normal epithelial cells and stromal cells, immune cells and vascular cells. This is the composition of the tumor's TME. The cells of the TME constitute an important part of the tumor tissues. Three TME phenotypes have been defined as per the genomic characteristics and clinicopathological features of GC [44]. They found TMEcluster-A was characterized by increases in the infiltration of cancer-associated fibroblasts and M2 macrophages. TMEcluster-B exhibited high infiltration of M0 macrophages and neutrophils. And TMEcluster-C showed increases in the infiltration of CD8+ T cells and M1 macrophages. Immune cell infiltration in the TME affects the prognosis of tumors [45, 46]. Immune cell infiltration has been determined as a biomarker for the diagnosis and prognosis of stage I-III colon cancer [47]. In our study, we determined that several immune-related responses showed a negative correlation with the risk scores, such as those by B cells, CD4+ T cells, CD8+ T cells, follicular helper T cells, and M1 macrophages (correlation coefficient < 0). However, M2 macrophages, cancer-associated fibroblasts, and endothelial cells were positively correlated with the patient risk scores (correlation coefficient > 0). Macrophages are the most abundant cells in the tumor matrix, and perform multiple functions in the TME [48]. The M2 macrophage phenotype reportedly has a tumor-promoting effect; in our study, this was positively correlated with the patient risk scores. In addition, this phenotype can influence multiple steps in the tumor development in other cells, including cancer-associated fibroblasts. However, the anti-tumor M1 phenotype has been shown to be a strong killer of cancer cells [48]. Tumor endothelial cells can accelerate tumor metastasis via tumor angiogenesis [49]. Thus, these findings can be further investigated for patients in the high-risk group, who presented with more severe cancer metastases. Immune checkpoint refers to a series of molecules that are expressed on immune cells and can regulate the degree of immune activation. They play an important role in preventing the occurrence of autoimmunity. Therefore, immune checkpoints are protective molecules in the body's immune system that act like brakes to prevent inflammatory damage caused by overactivation of T cells. But this mechanism is used by tumor cells, by suppressing immune cells. Thus, tumor cells can escape surveillance, or "immune escape". Thus, we showed the correlation coefficients between the 18-IRLP signature and the ICIs in Fig. 6F.

When we identified potential target genes, we look only at the correlation coefficient of lncRNA expression. Therefore, the top-ranked biological pathways almost have nothing to do with immune regulation or immune response. Here we want to look at another way in which these lncRNAs are involved in other important molecular pathways. A total of 27 immune-related lncRNAs were identified in our IRLP signature, and approximately half of them have not been reported in cancer. Among the ones that have been reported, LINC01232 promotes metastasis and participates in the progression of pancreatic cancer [50]. In GC, LBX2-AS1 positively regulates LBX2 mRNA stability, which affects the proliferation and apoptosis of GC cells [51]. LINC01082 expression is significantly downregulated in colon cancer tissues, and overexpression of LINC01082 can significantly suppress the proliferation of the colon cancer cells [52]. However, to date, no study has explored its biological functions in GC. In our molecular functional experiments, we found that overexpression of LINC01082 suppressed the invasion of GC cells. Moreover, the expression of PD-L1 was also suppressed, suggesting its role in tumor immunity. Above results suggest that LINC01082 may play a significant role in the development and progression of GC. As for other lncRNA in our signature, there are also some recent studies reported in cancers. For examples, VPS9D1-AS1 expression was shwn to be downregulated in GC tissues than that in adjacent non-tumorous tissues and its expression level was correlated with tumor size, TNM stage, overall and disease free survival [53]. Studies have proved that high expression of LINC02195 in human head and neck squamous cell carcinoma tissues and cell lines compared with normal mucosal tissues [54].

To conclude, our 18-IRLP signature had a strong and robust performance in predicting prognosis and immune response in GC. However, the limitations of our study must be acknowledged. First, the robustness of our signature was determined using gene expression; it should be verified in a larger sample size of patients with GC. Second, our 18-IRLPs gene signature included 27 immune-related lncRNAs. In our opinion, this number is high, and a prognostic signature with fewer lncRNAs can be built. Third, our model may help in the selection of ICIs and other immunotherapies, however, this would require further clinical trials. Finally, further experimental validation of these lncRNAs is required, not only including LINC01082.

## Conclusion

Based on bioinformatics and biological validation studies, we generated a novel 18-IRLP signature for prediction of prognosis and evaluation of immune responses in GC. Our IRLP signature provides novel insights into immunological biomarkers, and improves our understanding of the tumor immune microenvironment and therapeutic response in GC.

## Supplementary Information

**Additional file 1: Table S1.** The immune related pathway and genes.

**Additional file 2: Table S2. **The results of the univariate Cox proportional hazards regression model.

**Additional file 3: Figure S1. **The heatmap of all immune cell infiltration using various methods.

**Additional file 4: Figure S2. **The sensitivity of targeted drugs between high- and low-risk groups.

**Additional file 5: Table S3. **The target genes of these lncRNAs in our IRLPs signature.

**Additional file 6: Table S4.** The comparison of our IRLPs signature with other immune-related gene/lncRNA models in GC.

## Data Availability

All data generated or analyzed during this study are included in this published article and its additional files.

## Abbreviations

GC: Gastric cancer
lncRNA: Long non-coding RNA
TME: Tumor microenvironment
OS: Overall survival
TCGA: The Cancer Genome Atlas
IRLP: Immune-related lncRNA pair
FDR: False discovery rate
K-M: Kaplan–Meier
ROC: Receiver operating characteristic
AUC: Area under the curve
ICI: Immune checkpoint inhibitor
DMEM: Dulbecco’s modified Eagle’s medium
FBS: Fetal bovine serum
qRT-PCR: Quantitative real-time polymerase chain reaction
PBS: Phosphate-buffered saline
LASSO: Least absolute shrinkage and selection operator
